# Inhibition of STAT3- and MAPK-dependent PGE_2_ synthesis ameliorates phagocytosis of fibrillar β-amyloid peptide (1-42) via EP2 receptor in EMF-stimulated N9 microglial cells

**DOI:** 10.1186/s12974-016-0762-9

**Published:** 2016-11-21

**Authors:** Gen-Lin He, Zhen Luo, Ting-Ting Shen, Ping Li, Ju Yang, Xue Luo, Chun-Hai Chen, Peng Gao, Xue-Sen Yang

**Affiliations:** 1 Department of Tropic Hygiene, Institute of Tropical Medicine, Third Military Medical University, 30 Gaotanyan Street, Chongqing, 400038 People’s Republic of China; 2 Key Laboratory of Medical Protection for Electromagnetic Radiation Ministry of Education, Third Military Medical University, Chongqing, 400038 People’s Republic of China

**Keywords:** EMF, Microglia, PGE_2_, Synthesis, Phagocytosis

## Abstract

**Background:**

Prostaglandin E_2_ (PGE_2_)-involved neuroinflammatory processes are prevalent in several neurological conditions and diseases. Amyloid burden is correlated with the activation of E-prostanoid (EP) 2 receptors by PGE_2_ in Alzheimer’s disease. We previously demonstrated that electromagnetic field (EMF) exposure can induce pro-inflammatory responses and the depression of phagocytosis in microglial cells, but the signaling pathways involved in phagocytosis of fibrillar β-amyloid (fAβ) in microglial cells exposed to EMF are poorly understood. Given the important role of PGE_2_ in neural physiopathological processes, we investigated the PGE_2_-related signaling mechanism in the immunomodulatory phagocytosis of EMF-stimulated N9 microglial cells (N9 cells).

**Methods:**

N9 cells were exposed to EMF with or without pretreatment with the selective inhibitors of cyclooxygenase-2 (COX-2), Janus kinase 2 (JAK2), signal transducer and activator of transcription 3 (STAT3), and mitogen-activated protein kinases (MAPKs) and antagonists of PG receptors EP1-4. The production of endogenous PGE_2_ was quantified by enzyme immunoassays. The phagocytic ability of N9 cells was evaluated based on the fluorescence intensity of the engulfed fluorescent-labeled fibrillar β-amyloid peptide (1-42) (fAβ_42_) measured using a flow cytometer and a fluorescence microscope. The effects of pharmacological agents on EMF-activated microglia were investigated based on the expressions of JAK2, STAT3, p38/ERK/JNK MAPKs, COX-2, microsomal prostaglandin E synthase-1 (mPGES-1), and EP2 using real-time PCR and/or western blotting.

**Results:**

EMF exposure significantly increased the production of PGE_2_ and decreased the phagocytosis of fluorescent-labeled fAβ_42_ by N9 cells. The selective inhibitors of COX-2, JAK2, STAT3, and MAPKs clearly depressed PGE_2_ release and ameliorated microglial phagocytosis after EMF exposure. Pharmacological agents suppressed the phosphorylation of JAK2-STAT3 and MAPKs, leading to the amelioration of the phagocytic ability of EMF-stimulated N9 cells. Antagonist studies of EP1-4 receptors showed that EMF depressed the phagocytosis of fAβ_42_ through the PGE_2_ system, which is linked to EP2 receptors.

**Conclusions:**

This study indicates that EMF exposure could induce phagocytic depression via JAK2-STAT3- and MAPK-dependent PGE_2_-EP2 receptor signaling pathways in microglia. Therefore, pharmacological inhibition of PGE_2_ synthesis and EP2 receptors may be a potential therapeutic strategy to combat the neurobiological deterioration that follows EMF exposure.

**Electronic supplementary material:**

The online version of this article (doi:10.1186/s12974-016-0762-9) contains supplementary material, which is available to authorized users.

## Background

Electromagnetic field (EMF) exposure has been accelerated by technological advancements [1], increasing the health risks associated with neurological disorders, such as gliomas [2, 3] and Alzheimer’s disease (AD) [4, 5]. It is widely accepted that most cases of AD are associated with persistent inflammation and decreased clearance and degradation of amyloid beta (Aβ) [6, 7]. Additional evidence has revealed that pro-inflammatory cytokines act selectively to regulate the different types of microglial phagocytosis [8]. We previously observed pro-inflammatory responses and a depression of phagocytic activity in EMF-stimulated N9 microglial cells (N9 cells) [9]. However, it remains a central challenge to determine which special cytokines inhibit microglial Aβ clearance after EMF exposure.

Recent evidence confirmed the role of microglial pro-inflammatory responses in the development of AD [10]. Among the most important mediators of the prominent and rapid induction of the AD in the brain are cyclooxygenase 2 (COX-2) and prostaglandins [6, 11]. In particular, PGE_2_ is of interest in the development of AD, as it is initially significantly elevated in patients with very early stage or probable AD [12, 13]. Certain studies have shown that prostaglandin E_2_ (PGE_2_) mediates the potentiation of inflammatory responses and amyloid plaque formation [11, 14]. PGE_2_ can exert both detrimental and beneficial effects through four G-protein-coupled receptors named E-prostanoid (EP)1, EP2, EP3, and EP4 [15]. Among these, EP2 signaling is associated with pro-inflammatory gene up-regulation, the inhibition of beneficial chemokine production, and Aβ clearance underlying aging and/or Aβ_42_ accumulation [16]. Moreover, it has been revealed that PGE_2_ attenuates phagocytosis of latex beads by fibrillar β-amyloid peptide (1-42) (fAβ_42_)-stimulated microglia via the downstream EP2/protein kinase A (PKA) pathway [17, 18]. However, upstream pathways of PGE_2_ might also be important. Although much effort has been dedicated to identifying the upstream targets of COX-2-PGE_2_ cascades in microglia [19–22], the exact synthesis mechanisms of PGE_2_ underlying the salutary effect of EMF on microglial phagocytosis in AD remain largely unknown.

PGE_2_ plays an important role in the modulation of immune responses and inflammatory processes [23]. Three types of PGE synthases (PGESs) that participate in the synthesis of PGE_2_ have been described: one cytosolic PGES and two membrane-associated PGESs, microsomal PGES-1 and PGES-2 [24, 25]. Positive feedback by PGE_2_ on COX-2 and mPGES-1 expressions was previously observed and has received marked attention [26–28]. Recent evidence revealed the signaling pathways of the different regulation mechanisms of mPGES-1 and COX-2 and/or PGE_2_ release in primary activated rat microglia, involving protein kinase C (PKC), phosphatidylinositol 3-kinase (PI3K), IkB kinase (IKK)2, nuclear factor kappa-light-chain-enhancer of activated B cells (NF-κB), and mitogen-activated protein kinases (MAPKs) [19, 21]. Additionally, LPS-stimulated PGE_2_ release from spinal microglia is dependent on increases in COX-1 and/or COX-2 activity regulated by p38 MAPK activation [20]. In contrast, evidence from astrocytic cells has indicated a toxic role of Aβ_42_ in AD via a NF-κB-dependent mechanism [29]. In addition to these findings, the Janus kinase 2 (JAK2)/signal transducer and activator of transcription 3 (STAT3) pathway has also been reported to be involved in the inhibition of PGE_2_ production in BV-2 microglial cells [22]. We previously demonstrated a profound increase in the activated forms of JAK2 and STAT3 in EMF-stimulated N9 cells [30, 31]. These observations suggest that pharmacological inhibition of the JAK2-STAT3 and MAPK pathways in microglia may be a helpful approach in addressing the aforementioned questions.

Recent evidence suggests that an efficacious strategy against AD may be the promotion of phagocytosis and the inhibition of pro-inflammatory responses in microglia. Given the intricately intertwined role of microglia with the intracellular pathways involved in PGE_2_ production, in this study, we utilized a series of pharmacological approaches to investigate the molecular mechanisms that regulate Aβ_42_ phagocytosis-associated PGE_2_ synthesis in EMF-stimulated N9 cells. We demonstrated that JAK2-STAT3- and MAPK-dependent PGE_2_ synthesis are, at least in part, responsible for defective phagocytosis in EMF-stimulated microglial cells. The pharmacologic outcomes may provide critical information for targeting the microglial PGE_2_ synthesis in neurologic disorders that is associated with defective phagocytosis.

## Methods

### Cell culture and treatment

The immortalized murine microglial N9 cells were a gift from Dr. Yun Bai (Department of Genetics, Third Military Medical University, China) and were originally established by immortalization of day 13 embryonic brain cultures with the 3RV retrovirus carrying an activated v-myc oncogene [32, 33]. Cells were grown in Iscove’s modified Dulbecco’s medium (IMDM; HyClone, Logan, UT, USA) supplemented with 10% heat-inactivated fetal bovine serum (FBS; HyClone), 2 mM glutamine, 100 U/ml penicillin, 100 μg/ml streptomycin, and 50 μM 2-mercaptoethanol (Sigma-Aldrich, St. Louis, MO, USA). The cells were seeded in 25-cm^2^ T-flasks (5 × 10^6^ cells/flask), 6-well plates (5 × 10^5^ cells/well), and 24-well plates (1.5 × 10^5^ cells/well) at 37 °C in a humidified 5% CO_2_ atmosphere. N9 cells were passaged every 3 days at a 1:4 split ratio, and passages were used after 3–10 passages. After 24-h incubation, the cell culture medium was replaced with serum-free IMDM supplemented with or without the compounds of interest and incubated for 30 min prior to EMF stimulation. Then, cells were subjected to a 1-h process of phagocytosis 3 or 12 h after EMF exposure. Pharmacologic agents were purchased from Sigma-Aldrich unless otherwise indicated and were used in different experiments that included COX-2 inhibitor celecoxib (1, 5, 25 μM), JAK2 inhibitor AG490 (25 μM), STAT3 inhibitor S3I-201 (30 μM), p38 inhibitor SB203580 (10 μM), mitogen-activated protein kinase (MEK)-extracellular signal-regulated kinase (ERK) (MEK1/2-ERK1/2) inhibitor PD98059 (30 μM), c-Jun N-terminal kinase (JNK) inhibitor SP600125 (5 μM), antagonists of PG receptors EP1-4 (GW848687X (5 μM), AH6809 (10 μM), L-798106 (10 μM), GW627368X (10 μM; Cayman Chemical, Ann Arbor, MI, USA)), and a solvent control (tissue culture-grade dimethylsulfoxide (DMSO, 0.2%)). Doses of pharmacologic agents were chosen based on prior specificity studies and were shown to not alter the growth characteristics of N9 cells. None of the compounds displayed toxic or cytostatic effects in vitro at the corresponding concentration used in our experimental conditions (Additional file 1: Figure S1).

### Cell viability

N9 cells were indirectly assessed for cell viability using proliferation-based Cell Counting Kit-8 assay (CCK-8; Dojindo, Shanghai, China) 24 h after EMF exposure and aforementioned compounds treatment. Briefly, at the end of the culture period, 10 μl of CCK-8 solution was added to each well of the culture plate. After a 2-h incubation at 37 °C, absorbance at 450 nm was measured with a plate reader (BioTek Epoch, Winooski, VT, USA). A control was performed in parallel to monitor the influence of IMDM medium on the assays. Cell viability was expressed as a percentage of the control cell culture value using the following formula: cell viability = (absorption of sample − absorption of background)/(absorption of control − absorption of background) × 100%.

### Exposure system

As previously described, 2.45-GHz-pulsed microwaves, widely used in household appliances, medical applications, and communication systems, were employed [31]. Briefly, an EMF pulse was delivered through a rectangular horn antenna connected horizontally to a handset (Philips PM 7320X, Sivers IMA, Kista, Sweden). This system was set to deliver 90 mW through the handset. The pulse width was 2 μs, and the pulse repetition rate was 500 pulses per second. The cells were exposed to 2.45-GHz-pulsed microwaves for 20 min at an average specific absorption rate of 6 W/kg. During the 20-min exposure period, the handset was held over the culture layer of the flasks at a distance of 90 cm. For EMF exposure, the flasks were placed in the upper chamber of a Perspex^TM^ water bath (24.5 × 21 cm). The temperature of the medium in the flasks in the upper chamber was maintained at 37 °C by circulating heated water through a lower closed chamber. During sham exposure, flasks were placed in the same conditions but without EMF exposure.

### Phagocytosis assay

Phagocytosis was determined with fluorescent-labeled Aβ_42_ as previously described with slight modifications [17]. In brief, HiLyte^TM^ Fluor 647-labeled β-amyloid peptide (1-42) (AnaSpec, San Jose, CA, USA) was incubated at 37 °C for 7 days in a medium to promote fibril formation (647-fAβ_42_). Then, the 647-fAβ_42_ was diluted to 0.1 μg/ml with the medium and applied to N9 cells after the post-stimulation period (3 or 12 h) for either sham or EMF exposure. After 1-h incubation, a washing step with a cold serum-free medium was performed to interrupt any interaction between phagocytosing microglia and uningested 647-fAβ_42_. The phagocytic ability of the N9 cells was evaluated based on the fluorescence intensity of the engulfed 647-fAβ_42_ measured using a flow cytometer and fluorescence microscopy. In the fluorescence-activated cell sorting (FACS) analysis, the cells were collected and washed three times with ice-cold phosphate-buffered saline (PBS) and resuspended in 250 μl ice-cold PBS. The cell suspension was applied to a FACSVerse™ flow cytometer (BD Biosciences, San Jose, CA, USA).

For the morphological phagocytosis analysis, round glass cover slips were placed in the wells of 24-well plates before seeding N9 cells. Upon completion of phagocytosis, the glass cover slips were carefully taken out of the wells and rinsed twice in PBS. After being fixed and permeabilized, the cells were blocked with goat serum (Zhongshan Golden Bridge Biotechnology (ZsBio), Beijing, China) for 20 min at room temperature and washed three times in PBS. To label the membranes, cells were incubated with both the primary rat anti-mouse monoclonal antibody CD11b (1:200; AbD Serotec, Oxford, UK) and goat anti-rat Alexa Fluor 488 secondary antibody (1:500; Life Technologies, Carlsbad, CA) for 1 h at 37 °C with an interval step of three times wash. After washing and mounting, phagocytosis and microglial marker CD11b were visualized using a LSM 780 confocal laser scanning microscope (Carl Zeiss GmbH, Jena, Germany). Image analysis was performed based on a semi-quantitative method. Fluorescence intensity was measured using ImageJ 1.49 software.

### Enzyme immunoassay (EIA) of PGE_2_ and TNF-α

Cells were seeded in 6-well plates. After pharmacologic treatment and a 20-min EMF exposure of N9 cells in a culture medium, cell culture supernatants were collected and stored at −80 °C until use for detecting the level of PGE_2_ and TNF-α. The cells were washed three times with ice-cold PBS and resuspended in 100 μl ice-cold PBS. Ten-microliter aliquots of the cell collections were quantified using a cell counter (TC20, Bio-Rad, Hercules, CA, USA). PGE_2_ and TNF-α levels were quantified using an EIA kit (Cayman Chemical) and an ELISA kit (eBioscience, San Diego, CA, USA), respectively, according to the manufacturers’ instructions.

### Nitric oxide (NO) determination

The production of NO metabolites (nitrates and nitrites) in the culture medium was quantified using a NO detection kit (Nanjing Jiancheng Bioengineering Institute, Nanjing, China). Briefly, 100 μl of medium was added to each well. Then, 50 μl of nicotinamide adenine dinucleotide and nitrate reductase was added to each well. After 30 min, Griess reagents I and II (both 50 μl) were added and incubated for 10 min at room temperature. The optical density of each well was determined using a microplate reader with an emission wavelength at 540 nm.

### Quantitative real-time polymerase chain reaction

Cells were seeded in 6-well plates. After treatment, total RNA was isolated using TRIzol® regent (1 ml/well; Invitrogen, Carlsbad, CA, USA). RNA was reverse-transcribed using 1 μg total RNA and an oligo-dT primer using a PrimeScript™ RT reagent kit with a gDNA Eraser complementary DNA (cDNA) synthesis kit according to the manufacturer’s protocol (Takara Biotechnology, Dalian, China). Real-time quantitative RT-PCR analysis was performed using a Bio-Rad CFX Connect™ Real-Time PCR Detection System (Bio-Rad) and a KAPA SYBR® FAST qPCR kit (Kapa Biosystems, Boston, MA, USA). One-half microliter of cDNA of each sample and 0.2 μM of each primer were mixed in 20-μl reactions. Primers, *mouse COX-2* [NM_011198.3] forward 5′- GCTGGCCTGGTACTCAGTAGGTT -3′ and reverse 5′- CGAGGCCACTGATACCTATTGC -3′, *mPGES-1* [NM_022415.3] forward 5′- ACGACATGGAGACAATCTATCCT -3′ and reverse 5′- TGAGGACAACGAGGAAATGT -3′, and *EP2* [NM_008964.4] forward 5′- CCTTGGGTCTTTGCCATACT -3′ and reverse 5′- GCACTGGACTGGGTAGAACAG -3′ were designed and synthesized by Sangon Biotech Co., Ltd. (Shanghai, China). Primers hypoxanthine phosphoribosyl-transferase (*HPRT*) [NM_013556.2] forward 5′- GTTAAGCAGTACAGCCCCAAA -3′ and reverse 5′- AGGGCATATCCAACAACAAACTT -3′ were kindly provided by Dr. Xue Luo (Department of Tropical Physiology and Pathology, Institute of Tropical Medicine, Third Military Medical University, China). The PCR reaction conditions were as follows: 3 min at 95 °C for activation, 40 cycles of 3 s at 95 °C, and 20 s at 59 °C for COX-2, mPGES-1, and HPRT or at 63 °C for EP2 and HPRT, followed by 60–95 °C melt. The relative expression levels of COX-2 and mPGES-1 messenger RNAs (mRNAs) were normalized to an internal control HPRT using the 2^−ΔΔCt^ cycle threshold method [34].

### Immunoblot analysis

Cells were washed with ice-cold PBS and scraped in RIPA lysis buffer containing protease and phosphatase inhibitors (Roche, Penzberg, Germany). Whole-cell extracts (80 μg/lane) were separated using 10 or 12% SDS-polyacrylamide gel and then transferred onto PVDF membranes (Bio-Rad, Hercules, CA, USA). The membranes were blocked in PBS with 5% non-fat milk for 1 h and then incubated with their respective primary antibodies against COX-2 (1:200; Cayman Chemical), mPGES-1 (1:80; Santa Cruz Biotechnology, Santa Cruz, USA), and EP2 (1:200; Cayman Chemical), and with antibodies purchased from Cell Signaling Technology (Danvers, MA, USA) that recognize phospho-JAK2 Tyr-1007/1008 (p-JAK2, 1:1000), JAK2 (1:1000), phospho-STAT3 Tyr705 (p-STAT3, 1:1000), STAT3 (1:1000), phospho-p38 MAPK Thr180/Tyr182 (p38, 1:800), p38 MAPK (1:500), phospho-p44/42 MAPK (Erk1/2) Thr202/Tyr204 (1:1000), p44/42 MAPK (Erk1/2) (1:800), phospho-SAPK/JNK Thr183/Tyr185 (1:300), and SAPK/JNK (1:1000). The membranes were washed four times for 5 min each in Tris-buffered saline Tween-20 (TBST) and then incubated with horseradish peroxidase (HRP)-conjugated secondary antibodies (ZsBio) for 1 h at room temperature. After incubation, the membranes were reacted with enhanced chemiluminescence reagent (Bio-Rad), and the signal was detected using a ChemiDoc MP gel imaging system (Bio-Rad). Glyceraldehyde 3-phosphate dehydrogenase (GAPDH, 1:1000; Cell Signaling Technology) was used as an internal control. Relative band densities were determined by densitometric analysis using Image Lab software (Bio-Rad).

### Statistical analysis

Statistical analyses were performed using SPSS software. Each experiment was repeated a minimum of three times, and the data are expressed as the means ± SEM. The normality of the data were verified by the Kolmogorov-Smirnov test before further analysis. Significant differences between the groups were assessed by a one- or two-way ANOVA followed by Tukey’s test. Statistical significance was established at *P* < 0.05.

## Results

### Effect of EMF exposure on the phagocytosis of 647-fAβ_42_ in N9 cells

**Fig. 1:**
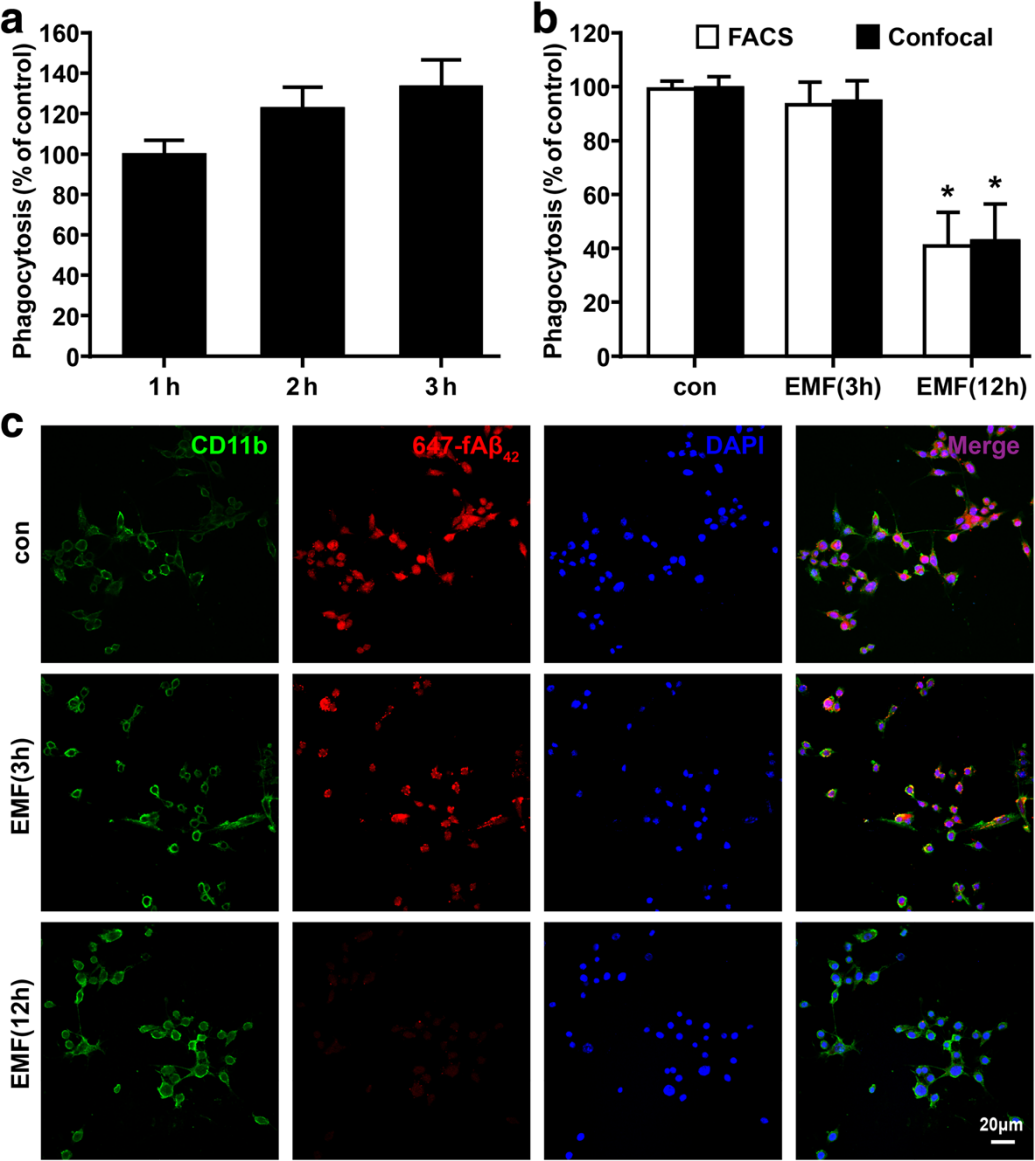
Flow cytometry and confocal microscopy analyses of the effect of EMF exposure on phagocytic capacity in N9 cells. N9 cells were exposed to 2.45-GHz EMF for 20 min. Untreated cultures were used as sham-exposed controls. N9 cells were subjected to a 1-h process of phagocytosis of fAβ_42_ with a strong fluorescent signal label HiLyte™ Fluor 647 (647-fAβ_42_) at the indicated time points after EMF exposure. **a** Continuous engulfment of 647-fAβ_42_ within 3 h in N9 cells. **b** Normalized average fluorescence intensity of 647-fAβ_42_ ingested per group estimated using a flow cytometer and a confocal microscope. *P < 0.05 vs the sham-exposed control group. **c** Microscopy images of 647-fAβ_42_ phagocytosis in N9 cells 3 and 12 h after EMF exposure. *Scale bar* = 20 μm

The fibril form of fluorescent-labeled Aβ is commonly used to mimic late-stage AD to investigate Aβ clearance [17, 35]. Given that Aβ_42_ fibrils enhance microglial phagocytic ability [8, 17, 18], low levels of fAβ_42_ and a strong fluorescent signal label HiLyte™ Fluor 647 were used in our experimental conditions. At a low concentration of 0.1 μg/ml, 647-fAβ_42_ was shown to be continuously engulfed within 3 h in the cultured medium (Fig. 1a). To avoid the probable duration and degradation of 647-fAβ_42_, a short 1-h phagocytic process was considered appropriate and was performed after EMF exposure in the following experiments. EMF was not found to cytotoxic to N9 cells 24 h after the 20-min exposure period (Additional file 1: Figure S1a). EMF resulted in decreased phagocytosis of 647-fAβ_42_ in the N9 cells after EMF exposure, as estimated via FACS (Fig. 1b). Compared to the phagocytic ratio in the sham exposure control cells (normalized to 100%), the ratio was slightly lower in cells at 3 h (94%) after EMF treatment, but the ratio decreased to 42% at 12 h after of EMF exposure (Fig. 1b). A similar result was observed via confocal microscopy, for which the fluorescence intensity was significantly decreased 12 h after EMF exposure (Fig. 1b, c). These findings suggest that EMF may be a potential risk factor for Aβ clearance in AD.

### Induction of COX-2-PGE_2_ synthesis is associated with phagocytic depression in EMF-stimulated N9 cells

**Fig. 2:**
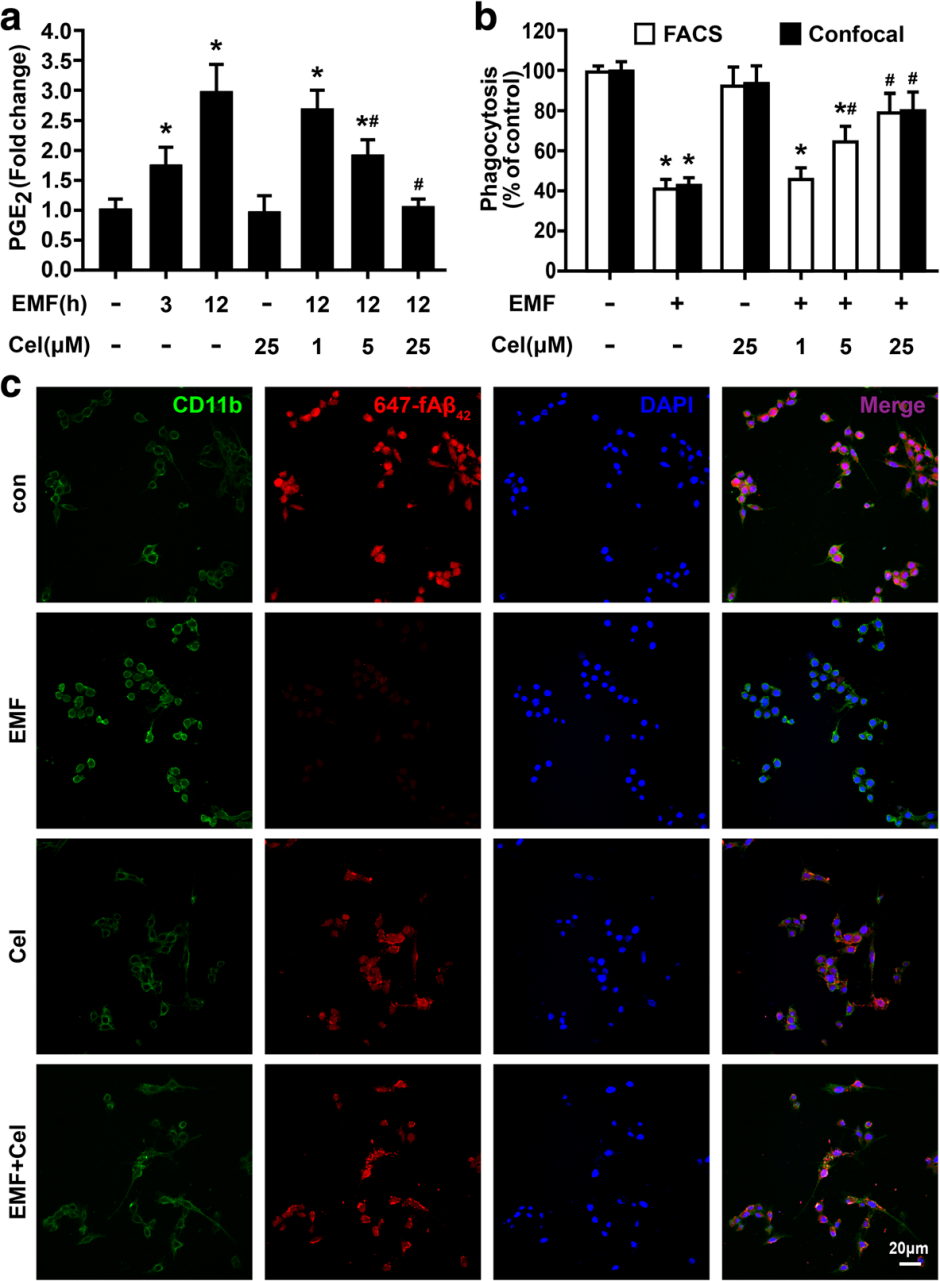
Improvement in phagocytic ability for EMF-exposed N9 cells with the addition of celecoxib. N9 cells were pretreated with or without celecoxib (1, 5, and 25 μM) for 30 min and then exposed to 2.45-GHz EMF (+) or sham exposed (−) for 20 min. Then, cells were subjected to a 1-h process of phagocytosis of 647-fAβ_42_ at the indicated time points after EMF exposure. **a** Enzyme immunoassay of PGE_2_ production in N9 cells pretreated with or without celecoxib 3 and 12 h after EMF exposure. **b** Normalized average fluorescence intensity of 647-fAβ_42_ ingested per group 12 h after EMF exposure estimated using a flow cytometer and a confocal microscope. For **a** and **b**, *P < 0.05 vs the sham-exposed control group; ^#^ *P* < 0.05 vs the EMF-exposed group. **c** Microscopy images of 647-fAβ_42_ phagocytosis in N9 cells pretreated with or without celecoxib 12 h after EMF exposure. *Scale bar* = 20 μm

Given the neuropathological features of AD, including the hallmark amounts of fAβ_42_ and PGE_2_ production [11–14], we examined the levels of PGE_2_ production in cell culture medium supernatants at the indicated times after EMF exposure. We found that the secretion of PGE_2_ was quite low in the sham-exposed control cells (Fig. 2a). EIA indicated that EMF exposure resulted in robust PGE_2_ release in a time-dependent manner and reached a maximum at 12 h (Fig. 2a). To investigate whether the induction effect of EMF on PGE_2_ release was related to the COX-2 and mPGES-1 enzymes, we determined the expressions of COX-2 and mPGES-1 and the production of PGE_2_ with or without the pretreatment with the selective COX-2 inhibitor celecoxib (1, 5, and 25 μM) in EMF-stimulated N9 cells. The production of PGE_2_ dramatically decreased in N9 cells cultured with celecoxib in a dose-dependent manner 12 h after EMF exposure (Fig. 2a). Furthermore, qRT-PCR and western blotting analysis showed that the levels of COX-2 and mPGES-1 were significantly increased 12 h after EMF exposure, and these increases could be suppressed by celecoxib pre-conditioning (Fig. 2c, d). We then tested whether the higher PGE_2_ release was associated with phagocytic depression after EMF exposure. The normalized phagocytic ratio was restored according to the concentration of celecoxib (1, 5, and 25 μM) for cells exposed to EMF compared with the sham-exposed controls (Fig. 2b, c), for which the phagocytic ratios were 46, 63, and 89%, respectively (Fig. 2b). Our findings confirm that EMF exposure can reduce phagocytosis of fAβ_42_ but that inhibition of PGE_2_ release using celecoxib can compensate for the reduction. These results indicated the involvement of PGE_2_ in the impaired microglial phagocytosis of fibrillar Aβ_42_ in response to EMF stimulation.

### Inhibition of STAT3- and MAPK-dependent PGE_2_ synthesis and phagocytic depression in EMF-stimulated N9 cells

**Fig. 3:**
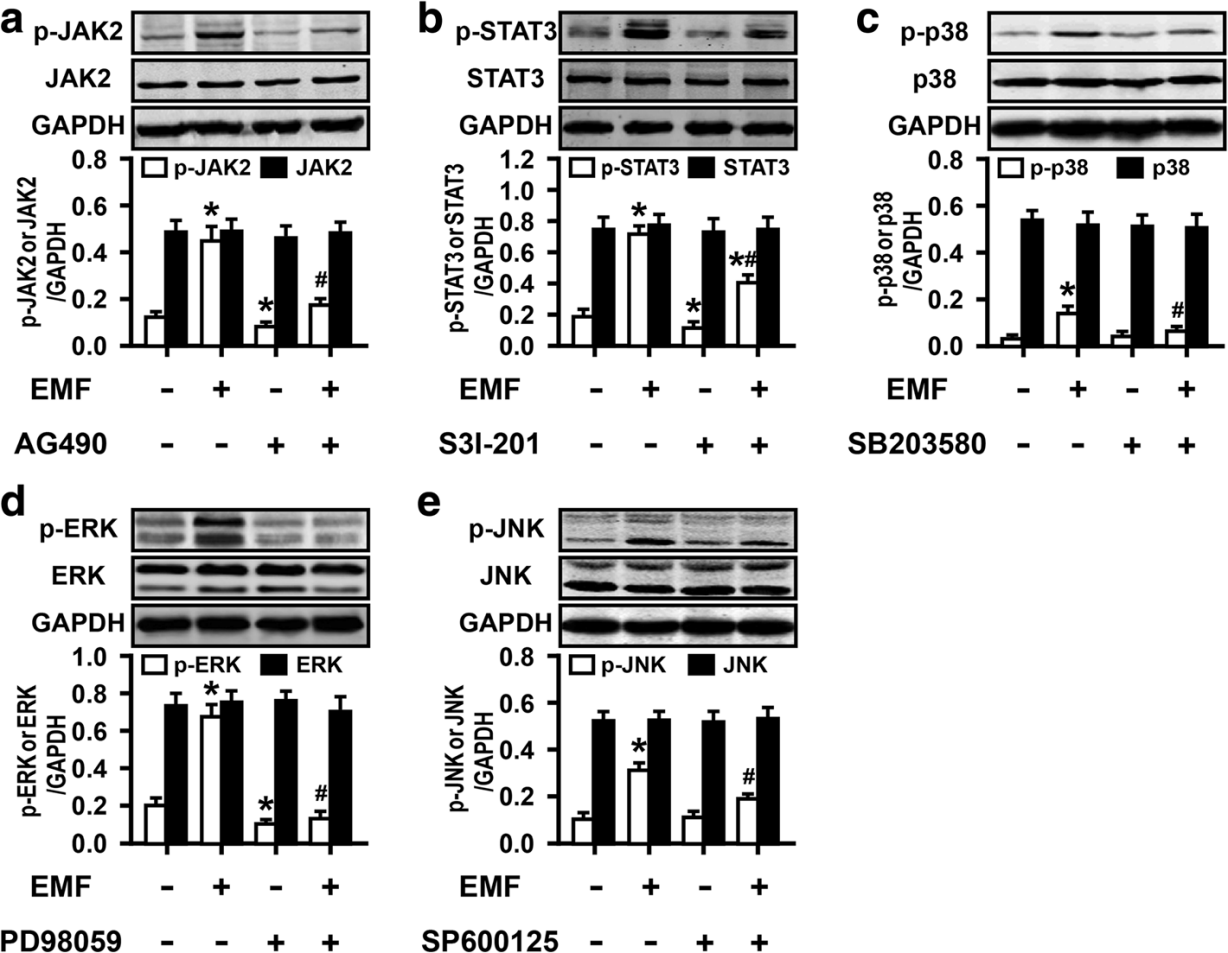
EMF exposure induces phosphorylation of JAK2, STAT3, and MAPKs in N9 cells. N9 cells were pretreated with or without JAK2 inhibitor AG490 (25 μM), STAT3 inhibitor S3I-201 (30 μM), p38 inhibitor SB203580 (10 μM), mitogen-activated protein kinase (MEK)-extracellular signal-regulated kinase (ERK) (MEK1/2-ERK1/2) inhibitor PD98059 (30 μM), c-Jun N-terminal kinase (JNK) inhibitor SP600125 (5 μM), for 30 min and then exposed to 2.45-GHz EMF (+) or sham exposed (−) for 20 min. The phosphorylation and expression of JAK2 (**a**), STAT3(**b**), p38(**c**), ERK1/2(**d**), and JNK(**e**) were determined, and the corresponding densitometric analyses were represented. *P < 0.05 vs the sham-exposed control group; ^#^ *P* < 0.05 vs the EMF-exposed group

**Fig. 4:**
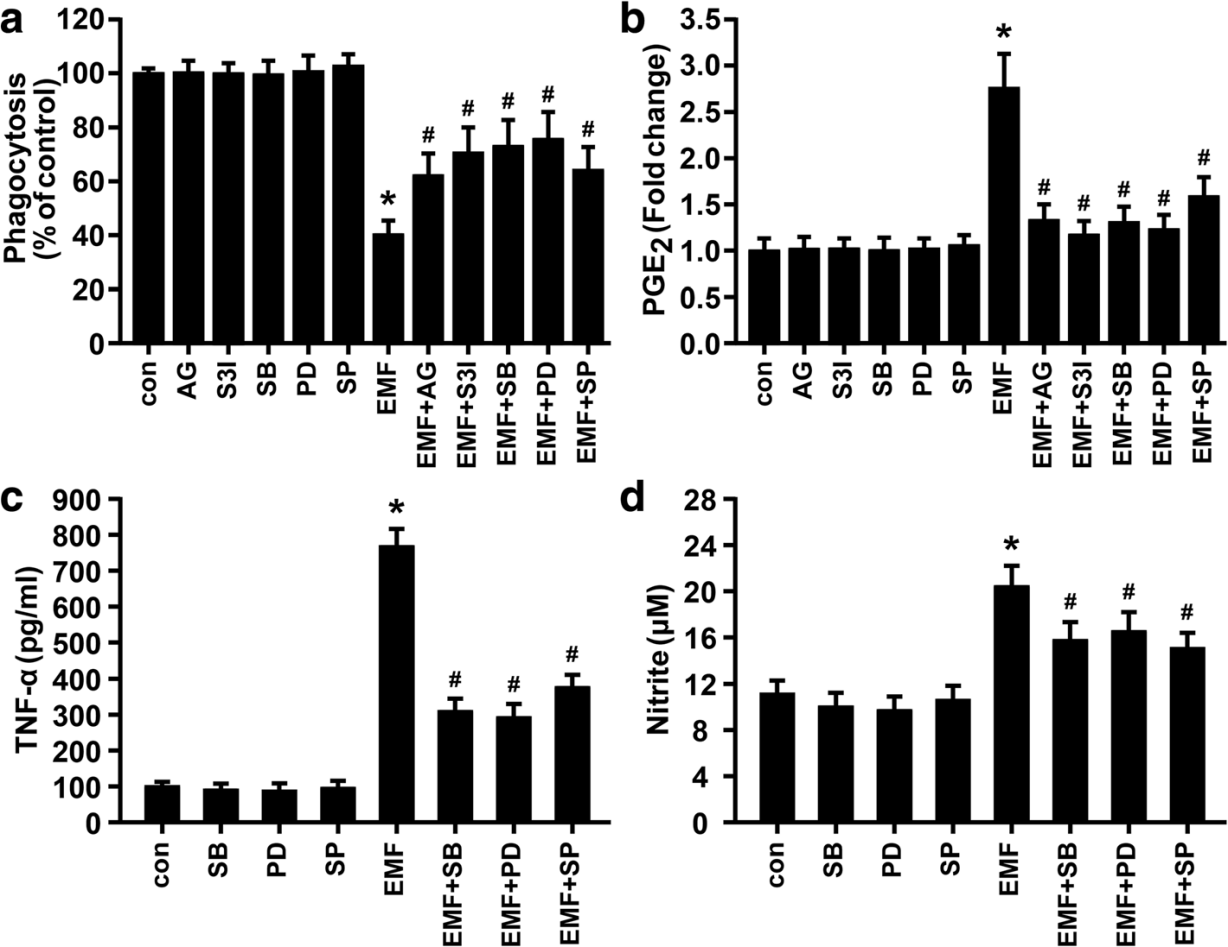
Inhibition of JAK2, STAT3, and MAPKs ameliorated the phagocytosis of 647-fAβ_42_ and abrogated the induction of TNF-α and NO in EMF-stimulated N9 cells. N9 cells were pretreated with or without celecoxib (25 μM), AG490 (25 μM), S3I-201 (30 μM), SB203580 (10 μM), PD98059 (30 μM), and SP600125 (5 μM), for 30 min and then exposed to 2.45-GHz EMF or sham exposed for 20 min. Then, cells were subjected to a 1-h process of phagocytosis of 647-fAβ_42_ 12 h after EMF exposure. **a** Normalized average fluorescence intensity of 647-fAβ_42_ ingested per group 12 h after EMF exposure estimated using a flow cytometer. Enzyme immunoassay of PGE_2_ (**b**) and TNF-α (**c**) production and Griess determination of nitrite (**d**) in N9 cells pretreated with or without the mentioned pharmacologic compounds of interest 12 h after EMF exposure. *P < 0.05 vs the sham-exposed control group; ^#^ *P* < 0.05 vs the EMF-exposed group

**Fig. 5:**
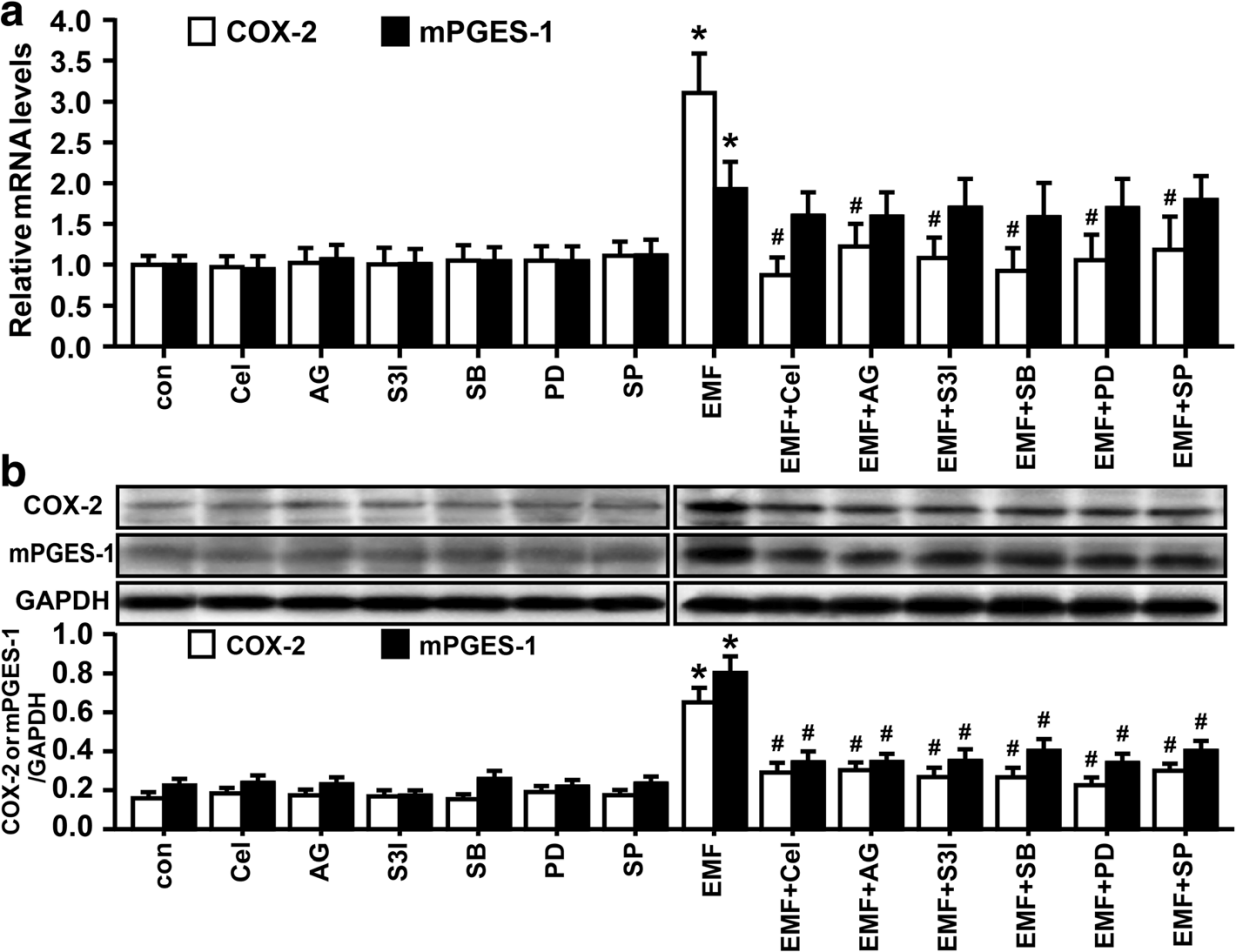
Involvement of JAK2, STAT3, and MAPKs in the regulation of the expression of COX-2 and mPGES-1 in EMF-stimulated N9 cells. N9 cells were pretreated with or without celecoxib (25 μM), AG490 (25 μM), S3I-201 (30 μM), SB203580 (10 μM), PD98059 (30 μM), and SP600125 (5 μM) for 30 min and then exposed to 2.45-GHz EMF or sham exposed for 20 min. Relative mRNA (**a**) and protein (**b**) levels of COX-2 and mPGES-1 in N9 cells pretreated with or without the mentioned pharmacologic compounds of interest 12 h after EMF exposure. *P < 0.05 vs the sham-exposed control group; ^#^ *P* < 0.05 vs the EMF-exposed group

To further analyze the candidate upstream signaling of COX-2-derived PGE_2_ synthesis, we first tested whether JAK2-STAT3 and MAPK signaling pathways are involved in the immunomodulatory action of PGE_2_ in EMF-stimulated N9 cells. Western blot analysis indicated significant phosphorylation of these signaling molecules in N9 cells after EMF exposure (Fig. 3a–e). It was also found that the cells treated with only the selective inhibitors of JAK2, STAT3, and MAPKs exhibited no change in phagocytic ability (Fig. 4a). Compared to the low phagocytic ratio of EMF-exposed N9 cells, depressed phagocytosis was abolished when the EMF-exposed N9 cells were pretreated with JAK2 inhibitor AG490, STAT3 inhibitor S3I-201, p38 inhibitor SB203580, MEK1/2-ERK1/2 inhibitor PD98059, and JNK inhibitor SP600125 (Fig. 4a). Not surprisingly, the production of PGE_2_ remained at the basal level when the cells were treated with only each inhibitor (Fig. 4b). In support of these, other pro-inflammatory factors TNF-α and NO release were dramatically suppressed by the inhibition of JAK2-STAT3 pathway in our previous study [30, 31]. In the present study, we also validated the inhibitory effects of TNF-α and NO production via MAPK inhibitors (Fig. 4c, d). Moreover, AG490, S3I-201, SB203580, PD98059, and SP600125 abrogated the phosphorylations of JAK2, STAT3, p38, ERK1/2, and JNK evoked by EMF stimulation (Fig. 3a–e), leading to the abolishment of PGE_2_ up-regulation caused by EMF exposure in N9 cells (Fig. 4b). We also found a similar suppression of COX-2 and mPGES-1 at mRNA and protein levels in N9 cells for pre-conditioning with these inhibitors, except for the mRNA level of mPGES-1 in the SP600125-treated cells, after EMF exposure (Fig. 5a, b). SP600125 did not significantly reduce EMF-induced mPGES-1 mRNA expression (Fig. 5a). Interestingly, SP600125 pretreatment led to a dramatic down-regulation of mPGES-1 protein in EMF-stimulated N9 cells (Fig. 5b), suggesting that the inhibition of mPGES-1 by indirect inhibition of JNK might occur at a post-transcriptional level. Overall, these data suggest that STAT3 and MAPKs may be the intracellular signal molecules mediating PGE_2_ action on phagocytic depression in EMF-stimulated N9 cells.

### EP2 is involved in PGE_2_-dependent phagocytic depression in EMF-stimulated N9 cells

**Fig. 6:**
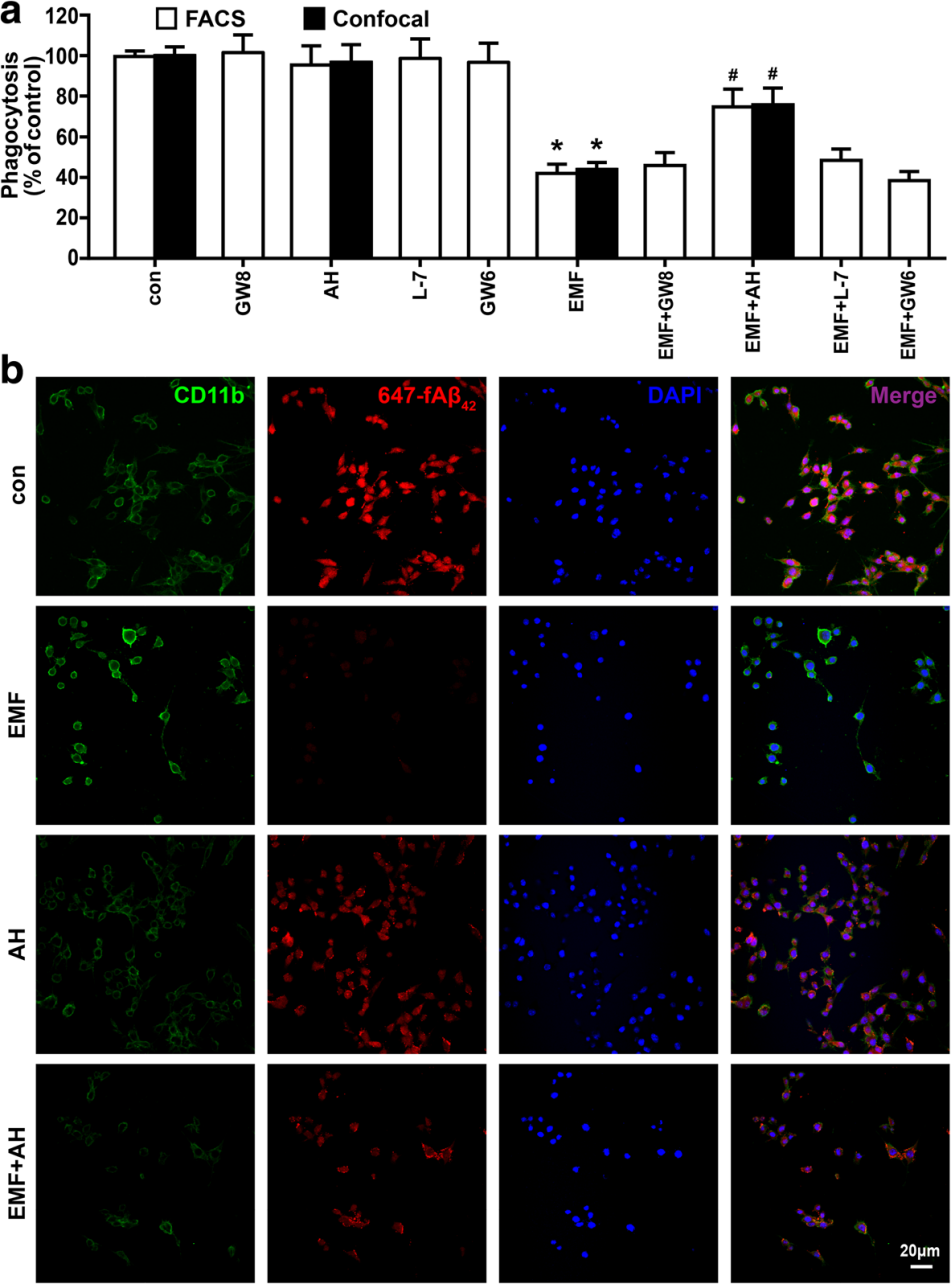
Involvement of EP2 activity in the restoration of impaired phagocytosis of 647-fAβ_42_ in EMF-stimulated N9 cells. N9 cells were pretreated with or without PG receptor EP1 antagonist GW848687X (5 μM), EP2 antagonist AH6809 (10 μM), EP3 antagonist L-798106 (10 μM), and EP4 antagonist GW627368X (10 μM). **a** Normalized average fluorescence intensity of 647-fAβ_42_ ingested per group 12 h after EMF exposure estimated using a flow cytometer and a confocal microscope. *P < 0.05 vs the sham-exposed control group; ^#^ *P* < 0.05 vs the EMF-exposed group. **b** Microscopy images of 647-fAβ_42_ phagocytosis in N9 cells pretreated with or without AH6809 12 h after EMF exposure. *Scale bar* = 20 μm

**Fig. 7:**
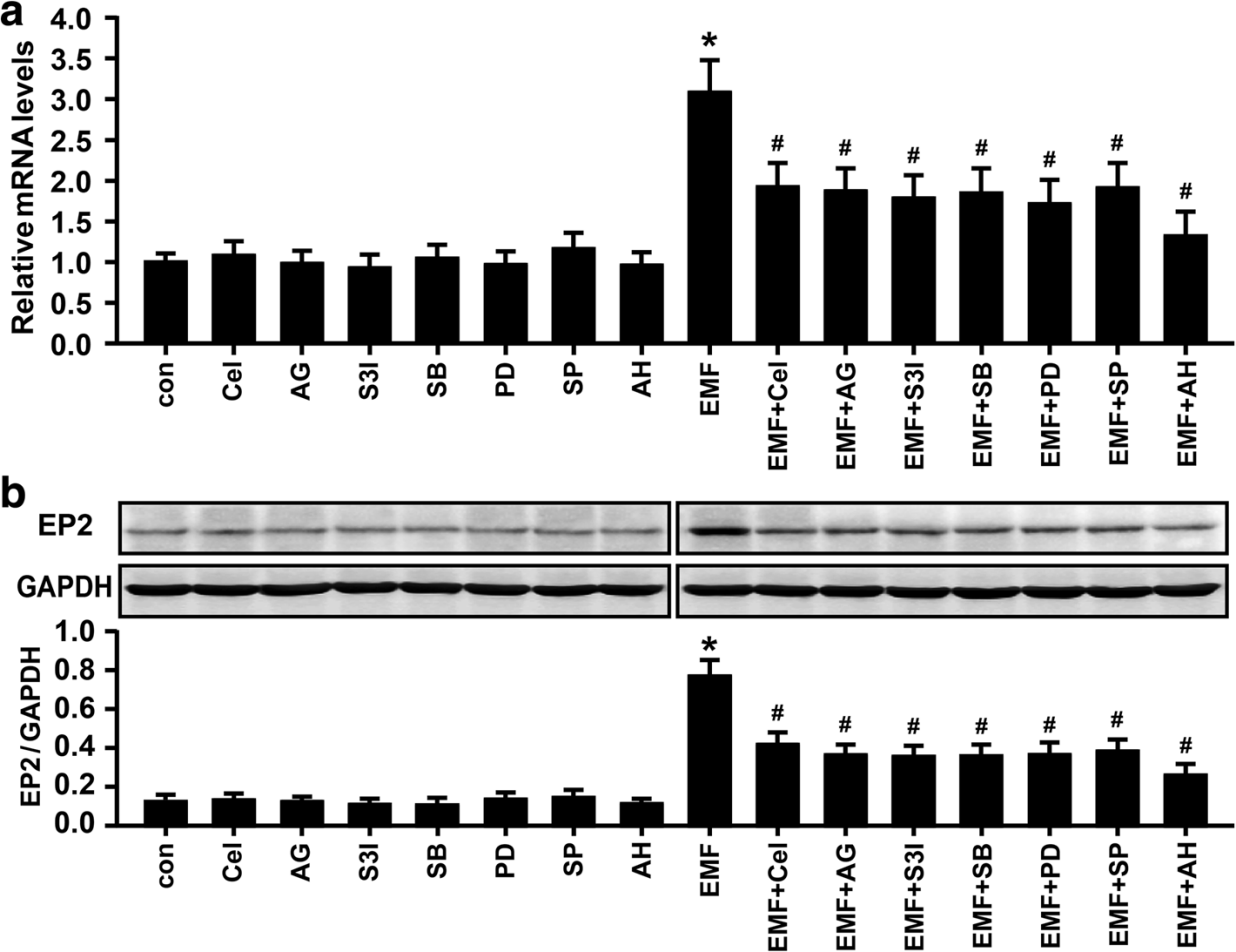
Involvement of COX-2, JAK2, STAT3, and MAPKs in the regulation of the expression of EP2 in EMF-stimulated N9 cells. N9 cells were pretreated with or without celecoxib (25 μM), AG490 (25 μM), S3I-201 (30 μM), SB203580 (10 μM), PD98059 (30 μM), SP600125 (5 μM), and AH6809 (10 μM) for 30 min and then exposed to 2.45-GHz EMF or sham exposed for 20 min. Relative mRNA (**a**) and protein (**b**) levels of EP2 in N9 cells pretreated with or without the mentioned pharmacologic compounds of interest 12 h after EMF exposure. *P < 0.05 vs the sham-exposed control group; ^#^ *P* < 0.05 vs the EMF-exposed group

In a previous work, PGE_2_-induced impaired microglial phagocytosis could be prevented by inhibiting EP receptors [18]. Thus, to identify the central receptor downstream of PGE_2_ signaling, we performed a phagocytosis assay with selective antagonists of EP1-4 in EMF-stimulated N9 cells. As shown in Fig. 6a, treatment with the EP2 antagonist AH6809, but not GW848687X (an EP1 antagonist), L-798106 (an EP3 antagonist), or GW627368X (an EP4 antagonist), significantly reversed the reduction effect of EMF-induced phagocytosis in N9 cells according to the FACS analysis. Clearly, the cells treated with AH6809, GW848687X, L-798106, and GW627368X alone showed no change in phagocytosis compared to the sham-exposed control cells (Fig. 6a). Confocal microscopy provided further evidence for the improvement in phagocytosis by AH6809 in EMF-treated N9 cells (Fig. 6b). Given the above results, we focused our studies on activation of the EP2 receptor by inhibiting COX-2, JAK2, STAT3, and MAPKs in EMF-stimulated N9 cells. We found that EMF exposure significantly increased the expression of EP2 (Fig. 7a, b). Moreover, the qRT-PCR and immunoblot analyses revealed that EP2 activation was blocked by celecoxib, AG490, S3I-201, SB203580, PD98059, and AH6809 in EMF-stimulated N9 cells (Fig. 7a, b). These results suggest that the PGE_2_-EP2 receptor signaling pathway might be involved in the JAK2-STAT3- and MAPK-mediated phagocytosis depression in N9 cells after EMF exposure.

## Discussion

In the present study, we observed a significant decrease in microglial phagocytosis of fluorescent-labeled fibrillar β amyloid (1-42) (647-fAβ_42_) and a distinct increase in endogenous PGE_2_ in N9 cells after EMF exposure. Inhibition of endogenous PGE_2_ production by a COX-2 inhibitor celecoxib improved phagocytic ability, which is consistent with our previous data, indicating that exogenous PGE_2_ depressed the fibrilled synthetic Aβ_42_-stimulated microglial phagocytosis of fluorescent-labeled latex beads [18]. Similar inhibitory regulation of mPGES-1 and COX-2 expressions and PGE_2_ release was observed after inhibition of various signaling pathways, including the JAK2-STAT3 and MAPK signaling cascades. Not surprisingly, these reduction effects due to the blockade of JAK2-STAT3 and MAPK signaling result in the amelioration of phagocytic ability in EMF-stimulated N9 cells. The inhibition study of E-prostanoid (EP) 1-4 receptors revealed evidence of the improvement in phagocytosis by the EP2 antagonist AH6809 in EMF-treated N9 cells. These findings are in agreement with other studies that have reported impaired phagocytosis in PGE_2_-related chronic inflammatory environments [11, 16]. Moreover, the inhibitors of COX-2, JAK2, STAT3, and MAPKs inhibited the expression of EP2. Combined with previous studies demonstrating that PGE_2_-EP2 signaling is related to impaired microglial clearance of Aβ and plaques [36–38], we suggest that EMF exposure could induce phagocytic depression via JAK2-STAT3- and MAPK-dependent PGE_2_-EP2 receptor signaling pathways in microglia.

It is well known that inflammatory activation of microglia and Aβ deposition are associated with the progression of Alzheimer’s disease (AD) [10, 14]. Several epidemiological and experimental studies have shown that EMF exposure can induce strong glial reactivity in different brain regions [39–41]. EMF exposure has also been demonstrated to increase the risk of AD [4, 5]. In a previous work, we demonstrated that an initial activation and pro-inflammatory response of microglia is induced by EMF exposure [30, 31]. Under the inflammatory conditions, we further observed an alteration in the clearance function of EMF-stimulated N9 cells [9]. Similarly, in this study, we observed an inversed correlation of pro-inflammatory COX-2 and prostaglandin expression with attenuated phagocytosis of 647-fAβ_42_ in EMF-stimulated N9 cells. In support of this, down-regulated phagocytosis of latex particles or apoptotic cells has also been observed in other members of the mononuclear phagocyte system, such as macrophages and monocytes, in the presence of static magnetic fields (SMFs) [42–44]. These decreased phagocytic activities were concomitant for driving the expressions of molecular components of various signaling pathways and effector functions, especially intracellular free Ca^2+^ ([Ca^2+^]_i_), in the presence of SMFs [42, 44]. In support of this, several studies emphasized a possible relationship between evoked levels of [Ca^2+^]_i_ and the synthesis of the classical pro-inflammatory mediator PGE_2_ [45, 46]. In contrast, contradictory evidence has indicated a significant increase in the phagocytic uptake of latex beads in macrophages after a short time of extremely low-frequency EMF [47, 48]. The heterogeneous regulation of phagocytosis by EMF may be largely attributed to the different parameters and times of exposure and to the different types of phagocytes, the different degrees of cellular differentiation or activation, and perhaps differences in potential inflammatory conditions. Thus, understanding the molecular pathways of the pro-inflammatory activation and the alteration of the clearance function in microglia triggered by EMF exposure might open new therapeutic options for suppressing AD development by improving Aβ-related microglial phagocytosis.

Recent studies have provided increasing evidence for the microglial pro-inflammatory responses and impaired microglial capacity of Aβ clearance in the brains of AD patients [8, 10, 49, 50]. It has been reported that microglia continue to produce pro-inflammatory cytokines but lose their Aβ clearance capabilities in a PS1-APP transgenic mouse model of AD [50]. Treatment with NO-generating compounds caused impaired phagocytosis in BV-2 microglia, and an inverse correlation between NO production and phagocytosis was observed upon β-amyloid pretreatment [51]. The addition of TNF-α reduces the expression of Aβ phagocytosis-related genes and decreases the uptake of Aβ in N9 cells [50]. Moreover, pro-inflammatory cytokines have been shown to inhibit fAβ-stimulated phagocytosis of fluorescent microspheres in BV-2 microglia [8]. In addition to down-regulating Aβ clearance pathways, pro-inflammatory cytokines (TNF-α, IL-1β, and interferon-γ) may also contribute to Aβ generation by up-regulating β-secretase and the active cleavage of APP [52, 53]. Considering the rapid induction of pro-inflammatory COX-2 and prostaglandins in the brains of AD patients [6, 11], we have previously reported the effect of PGE_2_ on the phagocytic ability of fAβ_42_-stimulated N9 cells and demonstrated a significant decrease in fAβ_42_-activated microglial phagocytosis of fluorescent-labeled latex beads by PGE_2_ [18]. In the present study, we observed a significant decrease in the microglial phagocytosis of 647-fAβ_42_ and a distinct increase in endogenous PGE_2_ in N9 cells after EMF exposure compared with the sham-exposed controls. Moreover, we found that incubation of cultured N9 cells with the COX-2 inhibitor celecoxib prevented the expression of mPGES-1 and COX-2, PGE_2_ release, and phagocytosis depression induced by EMF exposure. Taken together, these data suggested that pharmacological inhibition of the COX-2-PGE_2_ pathway may help critically improve microglia-targeted Aβ clearance against electromagnetic radiation.

Although PGE_2_ is of interest as the classic mediator of the inflammation process [16, 23], knowledge regarding the exact molecular mechanisms of the upstream regulators of PGE_2_ that underlie the intricate interplay in microglial pro-inflammatory responses and microglial phagocytosis of Aβ following EMF exposure is severely limited. Theoretically, mPGES-1 and COX-2 have been implicated in the inflammation-related biosynthetic pathway of PGE_2_ production [23]. In the present study, we validated that EMF exposure significantly increased mPGES-1 and COX-2 expressions and PGE_2_ release in N9 cells, which were accompanied by phagocytosis depression. In addition, uncoupled and heterogeneous patterns of mPGES-1 and COX-2 expressions have been revealed in LPS-activated primary rat microglia [19, 21]. To reveal the upstream regulators of the COX-2-PGE_2_ system, we investigated the involvement of the aforementioned inflammatory pathways. Our results demonstrated that different inhibitory levels of mPGES-1 and COX-2 mRNA and proteins were observed by pretreatment with pharmacological inhibitors of JAK2, STAT3, and MAPKs (p38, MEK1/2-ERK1/2, and JNK) in EMF-stimulated N9 cells. These inhibition studies also indicated the amelioration of the microglial phagocytosis of 647-fAβ_42_, reflecting the beneficial effects of JAK2-STAT3- and MAPK-dependent PGE_2_ blockages on the microglial phagocytic capacity after EMF exposure. Moreover, most previous studies have shown the involvement of the p38, ERK1/2, and JNK MAPK signaling pathways in the release of PGE_2_ from cultured microglia [19, 20], and macrophages have been shown to possess similar functional properties [54]. Moreover, STAT3 signaling was found to be critical in establishing the pharmacologic function of an active natural component, withaferin A, in LPS-induced PGE_2_ secretion from BV-2 cells and primary rat microglia [22]. Our results combined with previous studies suggest that microglial PGE_2_-mediated phagocytosis depression induced by EMF exposure is attributable in part to the activation of STAT3 and MAPK signaling.

As an important pro-inflammatory mediator, PGE_2_ exerts its actions locally through the binding of four molecularly and biochemically heterogeneous PGE receptors, termed EP1, EP2, EP3, and EP4 [15]. We previously investigated the key roles of EP1-4 in mediating PGE_2_ action in fAβ_42_-stimulated N9 cells using their specific agonists and antagonists, and we confirmed the involvement of EP2 in the regulation of microglial phagocytosis [18]. In this study, to reveal the essential role of microglial EP receptors in the action of PGE_2_-mediated inhibition of phagocytosis, we used EP antagonists to assess the improvement in microglial phagocytosis of 647-fAβ_42_. We found that the effect of PGE_2_ reduction on microglial phagocytosis of 647-fAβ_42_ appeared to be mainly mediated by EP2 in EMF-stimulated N9 cells. Our data corroborate other studies that have attributed microglial EP2 activity to the reduction of microglial clearance of Aβ and plaques using receptor-knockout animals [36–38]. Moreover, the qRT-PCR and immunoblot analyses showed significant abolishment of EP2 levels by the specific inhibitors of COX-2, JAK2, STAT3, and MAPKs used in our experimental conditions. Thus, these results suggest a potent role of JAK2-STAT3- and MAPK-dependent PGE_2_ in mediating phagocytosis depression via EP2 receptors in N9 cells after EMF exposure. Notably, differences exist between acute in vitro regulation of PGE_2_-mediated fAβ_42_ phagocytosis and chronic in vivo interactions between endogenous PGE_2_ and Aβ clearance from the entire cerebral cortex and hippocampus over several years. Although the up- and downstream immunomodulatory modes of PGE_2_ action and the known side effects have been widely described, the main pharmaceutical focus on the specific blockage of PGE_2_ synthesis and the inhibition of selected EP receptor activity remain to be solved.

## Conclusions

**Fig. 8:**
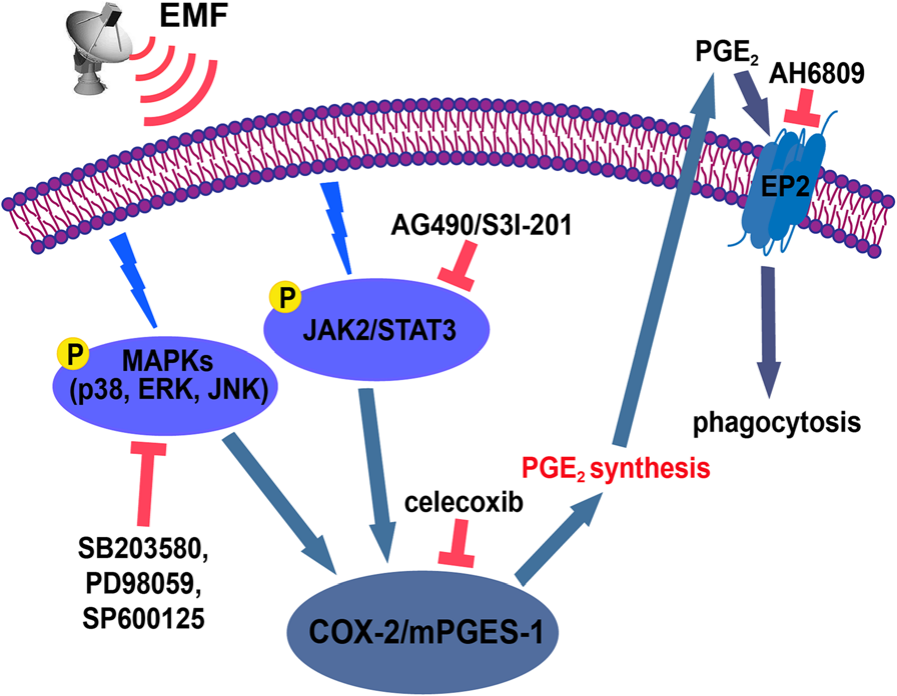
Schematic diagram illustrating the proposed immunomodulatory phagocytosis of fAβ_42_ via PGE_2_-related signaling mechanism in EMF-stimulated N9 microglial cells. External electromagnetic emission as a physical stimulation directly triggers an initial activation of microglia. Activation of JAK2-STAT3 and MAPKs signaling occurs in parallel with microglial activation, leading to PGE_2_ synthesis via COX-2-mPGES-1 system. Finally, PGE_2_ decreased microglial phagocytosis through EP2 receptor. Preventing phosphorylation of JAK2-STAT3 and MAPKs, inhibiting COX-2 activity, or abolishing EP2 activity efficiently ameliorated microglial phagocytosis during EMF stimulation

Our data provide detailed information regarding the intracellular signaling pathways involved in PGE_2_ synthesis and phagocytosis depression by EMF-activated N9 microglial cells (Fig. 8). Conversely, the selective inhibitors of JAK2, STAT3, and MAPKs ameliorated microglial phagocytosis via inhibition of the COX-2-PGE_2_ pathway. Simultaneously, these inhibitors attenuated EP2-related impaired microglial phagocytosis. These results suggest that JAK2-STAT3- and MAPK-dependent PGE_2_-EP2 receptor signaling modulates the phagocytosis of fAβ_42_ in EMF-stimulated microglia. Further studies targeting the combined use of pharmacological interventions of PGE_2_ and EP2 receptors will boost novel therapeutic approaches for AD and other neurodegenerative diseases.

## Additional file

Additional file 1: Figure S1. Effect of EMF and inhibitors on cell viability in cultured N9 cells. (a) N9 cells were exposed to 2.45 GHz EMF or sham exposed for 10, 20, and 30 min, and then cell viability was analyzed 24 h after EMF exposure. (b) Cell viability was measured after 24 h treatment of celecoxib (25 μM), AG490 (25 μM), S3I-201 (30 μM), SB203580 (10 μM), PD98059 (30 μM), SP600125 (5 μM), GW848687X (5 μM), AH6809 (10 μM), L-798106 (10 μM), and GW627368X (10 μM). (TIF 896 kb)

## Abbreviations

AD: Alzheimer’s disease
COX-2: Cyclooxygenase-2
EIA: Enzyme immunoassay
EMFs: Electromagnetic fields
EP: E-prostanoid
ERK: Extracellular signal-regulated kinase
FACS: Fluorescence-activated cell sorting
fAβ_42_: Fibrillar β-amyloid peptide (1-42)
HPRT: Hypoxanthine phosphoribosyl-transferase
JAK2: Janus kinase 2
JNK: c-Jun N-terminal kinase
MAPKs: Mitogen-activated protein kinases
MEK: Mitogen-activated protein kinase
mPGES-1: Microsomal prostaglandin E synthase-1
PGE_2_: Prostaglandin E_2_
qRT-PCR: Quantitative real-time polymerase chain reaction
STAT3: Signal transducers and activators of transcription 3
