# Supplementary figures and images for: Inhibition of STAT3- and MAPK-dependent PGE2 synthesis ameliorates phagocytosis of fibrillar β-amyloid peptide (1-42) via EP2 receptor in EMF-stimulated N9 microglial cells
Source: J Neuroinflammation. 2016 Nov 21;13:296. doi: 10.1186/s12974-016-0762-9 (PMC5117690; doi:10.1186/s12974-016-0762-9)

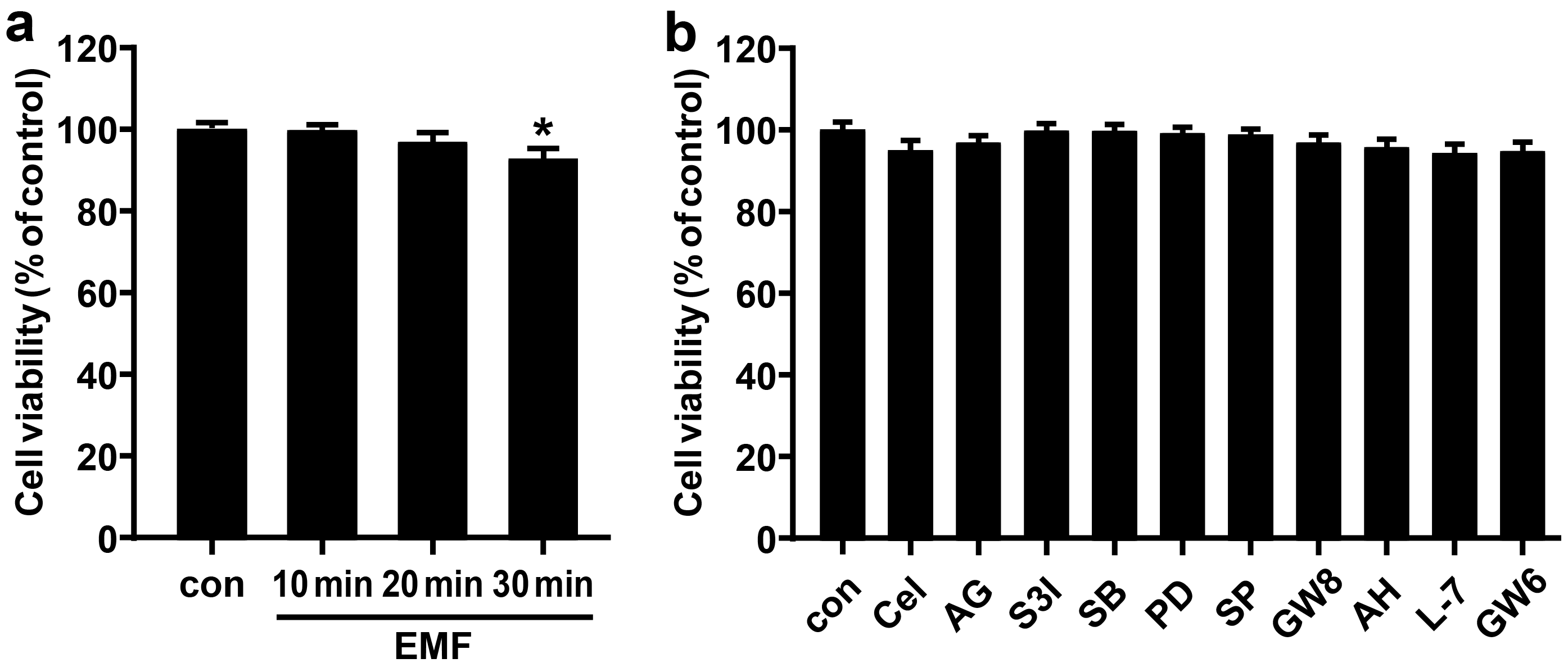

Supplement: Additional file 1: Figure S1. — Effect of EMF and inhibitors on cell viability in cultured N9 cells. (a) N9 cells were exposed to 2.45 GHz EMF or sham exposed for 10, 20, and 30 min, and then cell viability was analyzed 24 h after EMF exposure. (b) Cell viability was measured after 24 h treatment of celecoxib (25 μM), AG490 (25 μM), S3I-201 (30 μM), SB203580 (10 μM), PD98059 (30 μM), SP600125 (5 μM), GW848687X (5 μM), AH6809 (10 μM), L-798106 (10 μM), and GW627368X (10 μM). (TIF 896 kb) [file 12974_2016_762_MOESM1_ESM.tif]
